# Study protocol: a randomized controlled trial study on the effect of a game-based exercise training program on promoting physical fitness and mental health in children with autism spectrum disorder

**DOI:** 10.1186/s12888-018-1635-9

**Published:** 2018-02-27

**Authors:** Clare C. W. Yu, Simpson W. L. Wong, Farica S. F. Lo, Raymond C. H. So, Dorothy F. Y. Chan

**Affiliations:** 1 Department of Health and Physical Education, The Education University of Hong Kong, 10 Lo Ping Road, Tai Po, New Territories Hong Kong; 2 Department of Psychology, The Education University of Hong Kong, 10 Lo Ping Road, Tai Po, New Territories Hong Kong; 3 Department of Education Studies, Hong Kong Baptist University, Kowloon Tong, Kowloon, Hong Kong; 4 Hong Kong Sports Institute, Hong Kong. 25 Yuen Wo Road, Shatin, Hong Kong; 5 Department of Paediatrics, The Chinese University of Hong Kong, 6/F Clinical Science Building, Prince of Wales Hospital, Shatin, Hong Kong

**Keywords:** Autism Spectrum Disorder, Game-based learning, aerobic exercise, Physical health, Psychological well-being

## Abstract

**Background:**

Suboptimal physical activity levels and tolerance, poor motor skills and poor physical health are demonstrated in children with Autism Spectrum Disorder (ASD). We speculate that social interaction and communication deficits in children with ASD are two major factors that hinder these children from actively participating in group physical activities. While previous studies have demonstrated that exercise intervention improves motor skills and behavioral outcomes in children with ASD, these programs tend to focus only on a single sport, which may not cater to the interests of different children with ASD. In this protocol, a game-based exercise training program designed by a multi-disciplinary team (pediatrics, physical education and psychology) will be implemented by front-line healthcare providers trained following the train-the-trainer (TTT) model and subjected to validation.

**Method:**

Using a randomized controlled trial design, the effectiveness of the game-based exercise program will be examined for 112 young children with ASD. These children were randomly assigned to two groups, which will be tested and trained in either one of the two arms of the waitlist conditions (control and intervention). The assessment of physical and psychological traits will be conducted at baseline (pre-test), at 16-weeks (post-treatment) and at 32-weeks (follow-up) of the program.

**Discussion:**

Most of the interventions designed for ASD children target either their psychological traits or physical conditions, without bridging the two states. With the recognition of bidirectional relations between mental and physical health, the present game-based exercise program which includes multiple level of difficulties was developed to equip ASD children with the necessary skills for engaging in sustainable team sports or even professional sport training. The program, if effective, will provide an entertaining and engaging training for whole-person development among children with ASD.

**Trial registration:**

This study is registered with the Chinese Clinical Trial Registry (ChiCTR-IOR-17011898). Registered 6^th^ July 2017.

## Background

The prevalence of Autism Spectrum Disorder (ASD) is increasing globally, and Hong Kong is of no exception [1]. In Hong Kong, the average prevalence of ASD was 16.1 per 10000 for children less than 15 years old from 1986 to 2005 [2]. Since then, new cases of ASD in Hong Kong children have increased remarkably, with 184% new cases reported from year 1997 to 2005 [3]. ASD is characterised by a number of neurodevelopmental-based impairments, including social interaction and communication deficits, repetitive and stereotypical behaviours and interests [4]. Apart from the core diagnostic symptoms, many ASD children may also have impairments in the cognitive-behavioural and perceptuomotor domains, motor function, as well as static and dynamic balance [5]. Because of these social and behavioural deficits, children with ASD typically present with decreased physical activity levels, sedentary lifestyle, and lower exercise tolerance relative to their typically developing counterparts [6, 7]. Motor skills in ASD children are typically poorly developed or delayed [8], and sadly, ASD in children is often comorbid with developmental coordination disorder [9]. These deficiencies in motor control, coupled with a lack of engagement in physical activity, make individuals with ASD prone to chronic diseases [10]. Obesity, for example, is highly prevalent amongst children with ASD [5, 10].

Five previous reviews have been conducted regarding the impact of physical activity or exercise intervention on children and adults with ASD [11–15] and all report promising results. Apart from improvements in motor skills, improvements on numerous behavioural outcomes including stereotypic behaviours, social-emotional functioning, cognition and attention have also been reported in children after physical activity or exercise intervention [12, 13]. Vigorous exercise was reported to have a more pronounced effect than milder less strenuous exercise [12, 16–19]. However, studies that use a randomized controlled trial (RCT) design [20–24] or focus on younger children (4 to 6 years old) [25] are very limited. Among the existing studies with children, jogging and swimming are the two most common exercises selected as the core component of the intervention. Other exercise items include horseback riding, martial arts, resistance training, yoga and dance [12, 13].

To encourage children with ASD to engage in physical activity, more support for these children and their parents is necessary. The present study implements a multidimensional training program that incorporates elements such as aerobic and resistance training, fundamental movement skills, speed, plyometric and agility development, etc. to benefit these children, especially the younger age group [26]. The multidimensional design of the exercise training program aims to strengthen the fundamental physical conditions that support a variety of sports and exercise activities. The incorporation of multiple sport skills also prevents the risk of overuse injuries when performing a single exercise training in the long run [26]. To the best of our knowledge, no studies of exercise training program with multidimensional training elements in young children with ASD have been published. Thus, our research team consisting of experts from multiple disciplines such as pediatrics, psychology, sport sciences and physical education designed the present game-based exercise program to provide more comprehensive support for children with ASD. Furthermore, we adopted a train-the-trainer (TTT) model to facilitate the sustainable use of the program in the community. Following this model, we trained a group of front-line healthcare providers from non-governmental organizations, who in turn implemented the training on children.

The training program in the present study was designed specifically to cater to children. The exercise activities were presented to children in the form of games, with each game carefully designed such that it has elements that train both physical and mental abilities. In addition, the games were designed to include various levels of difficulty so that trainers can administer the game at appropriate levels for children. This may also motivate children to progress to higher levels in the course of the training. In this study proposal, the impact of our exercise program on the physical and psychological conditions of children with ASDs was hypothesized. The success of the program will also provide support for the feasibility of the train-the-trainer model.

### Aims and hypotheses

The first aim of this study was to test the effectiveness of a supervised game-based exercise training program in promoting motor skills and physical fitness, as well as reducing stereotypical and maladaptive behaviours in preschool children with ASD. These physical abilities served as the primary outcomes, while social and language skills were measured as secondary outcomes. Secondly, this study also aimed to evaluate the efficacy of this program as implemented by front-line healthcare providers through the train-the-trainer approach. We hypothesized that when compared to the waitlist control group, the exercise training program will lead to (1) increased motor skills and physical fitness and reduced stereotypical and maladaptive behaviours in preschool children with ASD; thus providing evidence for (2) the feasibility and sustainability of this supervised tailor-made exercise training program for young children with ASD following the train-the-trainer approach.

## Methods/Design

### Study design

**Fig. 1:**
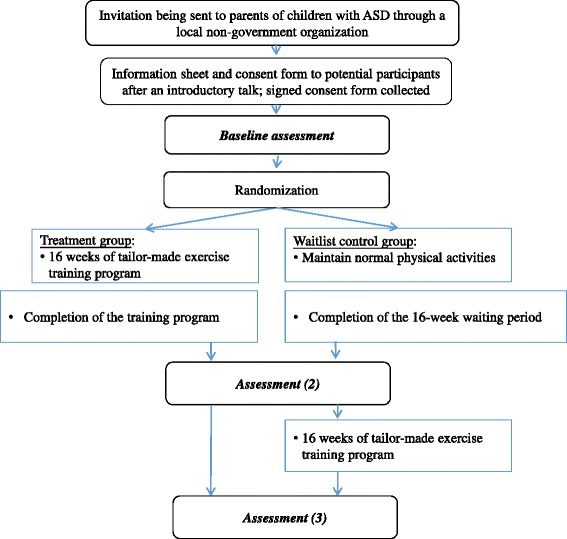
Flow chart of the study design

This is a randomized controlled trial (RCT) study registered with the Chinese Clinical Trial Registry (ChiCTR-IOR-17011898). The study is ongoing and is during the subject recruitment phase (August to Sept, 2017). Community centers within a local non-government organization were recruited. The recruited ASD children will be randomly assigned to either the treatment group or a wait-list control group using the *simple randomization* method with the employment of a toolbox provided in MS Excel, to be performed by the first author. The exercise training program will be offered to children in the wait-list control group after the posttest of the treatment group (Fig. 1).

### Participants

With an agreed partnership with a local non-governmental organization (NGO), children were recruited through the community centers within this NGO. Front-line healthcare providers (mainly social workers) from these centers was recruited as trainers to implement the supervised exercise training program for children with ASD after completing the train-the-trainer workshop. Children aged 4 to 6 years old with a formal diagnosis of either Autism, Autistic features, Autism Spectrum disorder, Asperger Syndrome, Pervasive Developmental Disorder by Developmental Paediatricians or Clinical Psychologists based on the Fourth Edition of the Diagnostic and Statistical Manual of Mental Disorder (DSM-IV) were invited to join the study[27]. Children with underlying congenital abnormalities or other diseases that limit them to participate in daily physical activities was excluded from the study.

### Intervention

The exercise program is a 16-week game-based exercise training program. Two sessions will be run each week to form a total of 32 sessions over 16 consecutive weeks. Each session will last for an hour. Six to eight stations will be set up and children will be instructed to finish the exercises in all stations one after another in a fixed order. After finishing a circuit of the prescribed exercises in a particular session, children will be guided by front-line healthcare providers to complete another circuit of the prescribed exercises. Three circuits of prescribed exercises are expected to be finished within a training session. The exercises in different stations are designed to train various muscle groups or fitness components. Exercise stations within an exercise session will be changed biweekly according to the children’s progression. Parents of the participating children are encouraged to join the class and accompany their child during the training session.

The program encapsulates physical, social, linguistic and communication skills training aspects, and can be divided into three phases (about 5 to 6 weeks for each phase) to achieve different targets:

#### Phase I

To establish trust between the children and their coaches, and to allow children to get used to the training regime of the program. Paired group activities are included in this phase.

#### Phase II

To promote cardiopulmonary fitness and muscular strength, and to increase the range of movement of large joints. Paired group activities are included in this phase, with increased exercise intensity of each session.

#### Phase III

To reduce maladaptive behaviors amongst the children and to improve their attention span. Exercise intensity for each session is the highest among three phases. Large group activities are included in this phase.

### The train-the-trainer approach

**Fig. 2:**
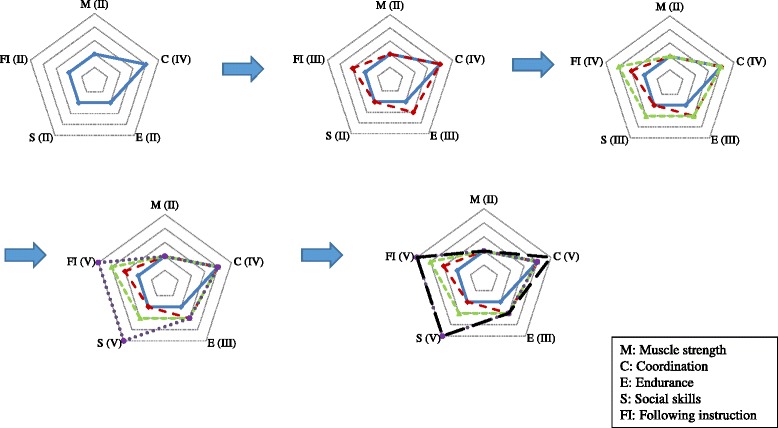
An example of the radar charts showing the progression of the rope skipping exercise from the beginning stage (simple, play by themselves) to the final stage (complex, needs interaction with other children)

Two 4-hour sessions of the train-the-trainer workshops will be provided for front-line healthcare providers participating in the study. The workshop a) covers the developmental characteristics, medical issues and special needs in ASD children, b) discusses the basic concepts, training skills and safety issues of exercise training for children with ASD, after which c) the details of the 16-week exercise training program will be introduced. Steps for running the exercises and using the exercise equipment correctly will be demonstrated by professional trainers. The workshop also involves a trial run of the exercises among the participants. We further prepare a manual that provides information on the method and training goals, exercise and visual tools, safety concerns, and guidelines for adjusting the difficulty levels of each exercise to current physical fitness levels of the participants. In addition, five physical and psychosocial components including muscle strength, coordination, endurance, social skills, and following instruction should also be evaluated and plotted on a radar chart for each exercise item (Fig. 2). This information will facilitate front-line health care providers to select and plan for their exercise training sessions for their group of children. Each training class will be conducted by three front-line healthcare providers with a group of 7 to 8 children in each community center. A professional coach and an exercise specialist in our research team will visit each center biweekly during the 16-week training period to observe the progress of the training classes and provide feedback to the front-line healthcare providers for the progression of the training sessions.

### Ethics

The study protocol has been approved by the Human Research Ethics Committee of the Education University of Hong Kong (Ref. No.: 2016-2017-0223). All the participants will join the research on a voluntary basis. After an introductory presentation at each community center, an information fact sheet and a consent form will be distributed to the front-line healthcare providers and parents. The completed consent form will be collected by the research team before the study commences. Consent to publish will also be obtained from parents that information obtained from this research may be used in future research and may be published, however, the personal details of each child will not be revealed; videotape or photos of the training sessions may be taken which will be used for teaching or presentation purpose, however, children’s’ faces will be blurred on the videos / photos and cannot be recognized.

### Measurements

#### Symptoms of ASD

##### The Childhood Autism Spectrum Test (CAST)

Previously known as the Childhood Asperger Syndrome Test, is a 37-item parental questionnaire to screen for Autism Spectrum Disorders [28]. The cut-off score is 15 for possible ASD or related social-communication difficulties.

##### The Autism Spectrum Quotient-Children’s Version (AQ-Child)

Is a parent-report questionnaire to quantify autistic traits [29]. There are five subscales. A Chinese version was developed in 2008 [30].

#### Children’s physical fitness

Children’s physical fitness will be assessed by a battery of tests: the PREFIT battery [31], which comprises the measurements of body weight and height and waist circumference to assess anthropometry, the PREFIT 20 meter shuttle run test [32] for cardiorespiratory fitness, handgrip strength and standing long jump tests for muscular strength of upper limbs and lower limbs respectively, 4 x 10 meter shuttle run test for speed-agility and one-leg stance test for balance [31]. In addition, the upper limb power [33] of each child will be assessed.

##### Anthropometric

Standing height without shoes will be measured using a seca 217 stable stadiometer to the nearest 0.1 cm. Body weight will be measured using a portable Tanita scale (Model BF-522; Tanita Corporation, Tokyo, Japan). Waist circumference is taken as the mean of two readings of the minimum circumference between the umbilicus and xiphoid process.

##### ***PREFIT 20 meter shuttle run test*** [32]

In this test, children run back and forth between 2 parallel lines 20 meters apart concurrently with an audio signal. The test finishes when the child failed to reach the end lines concurrent with the audio signal on two consecutive occasions or when the child stops because of exhaustion. Because of the young age of the preschoolers, adaptations of the original test will be made by decreasing the initial speed and by having two evaluators running with a reduced group of children in order to provide an adequate pace [31]. Test results will be expressed as the number of laps completed.

##### Handgrip strength

Each child will be given a brief demonstration and verbal instructions for the handgrip strength test using the Takei T.K.K.5001 GRIP-A handgrip dynamometer (Takei Scientific Instruments Co. Ltd, Tokyo, Japan). The dynamometer will be adjusted according to the child’s hand size. The test will be done in the standing position, with the wrist in the neutral position and the elbow extended. Children will be given verbal encouragement to ‘squeeze as hard as possible’ and apply maximal effort for at least 2 seconds. Two trials will be allowed alternately with both hands. The best value (kg) of the two trials for each hand will be chosen, and the average of both hands will be recorded [31, 34].

##### Standing broad jump

After demonstration, the child will be instructed to stand with his/her feet at the shoulder’s width, and toes just behind the take-off line. When ready, he/she bends the knees with swing both arms, then push off vigorously and jump as far as possible. The distance is measured from the take-off line to the point where the back of the heel nearest to the take-off line lands on the ground [35]. Three jumps are allowed and the best of these attempts will be recorded [31].

##### 4 x 10 meter shuttle run

Two parallel lines 10 meters apart will be marked on the floor using marking tape. Children will be instructed to run and turn as fast as possible between the two lines. The test covers a distance of 40 meters. The best of two attempts will be recorded in seconds [31].

##### One-leg stance test

In this tests, the child stands on one foot with the supporting leg on the floor and the free leg flexed at the knee, maintaining the balance position as long as possible. The test ends when the child cannot maintain the required position. The test will be done once with each leg and the mean time (seconds) will be used in the analysis [31].

##### Overhand throw

When ready, the child uses the preferred arm to complete an overarm throw of a bean bag (140 grams). Three trials are allowed and the furthest distance for the throw will be recorded in centimeters [33].

#### Children’s nonverbal intelligence, socio-emotional skills and cognitive-linguistic skills

##### The Standard Raven’s Progressive Matrices (SRPM)

This is a test of nonverbal reasoning ability [36]. The Standard Raven’s Progressive Matrices (SRPM) consists of 60 patterned diagrams with a missing part to be identified. The *SRPM is norm-referenced in Hong Kong*. This test will be used to estimate nonverbal ability among our participants.

##### The Chinese version of the Psychoeducational Profile, Third Edition (CPEP-3)

The CPEP-3 assesses skills and behaviors in children with ASD [37]. We will administer the social reciprocity and affective expression subtests. *The Psychoeducational Profile-Revised (PEP-R)* has been previously translated into Chinese and a validation study done in Hong Kong to examine the psychometric properties [38]. The current Chinese PEP-3 edition is also well validated and will be used in this study [39].

##### Joint attention task

The assessment of joint attention for socio-emotional skills will be performed under supervision of the experimenter. A series of tasks will be conducted within a time span of 25 minutes. The tasks are based on earlier developed tasks to provoke checking, gazing, pointing, follow pointing and joint visual attention of the child [40].

##### The face and eye tests

The ability to read facial expressions and eyes for the understanding of emotion is assessed by the *Face and Eye tests* developed by a team of researchers from the Autism Research Centre, University of Cambridge [41, 42]. The two tests have been widely used for assessing children with autism spectrum condition.

*The Faces Test* is a 20-item test on simple emotions and complex mental states [41]. Originally, the photos were taken with a western model. As our project takes place in Hong Kong where all our participants are Chinese, a Chinese model was recruited to pose the same facial expressions. The photographs were displayed digitally with the corresponding emotion or mental state word below the photo. The translation of those emotions was completed by independent translators and all discrepancies were resolved by consensus.

*The Eyes Test* is a 28-item test with photos of different sets of eyes belonging to different people [42]. The test is designed to examine social intelligence by investigating how well an individual can identify the photographed person’s feelings or thoughts. This is more challenging than the Faces Test, as participants have to choose among four options provided, the feelings and thoughts are more implicit, and the eyes are the only available clue to determining the person’s emotion.

##### Recognition of facial emotions

For the facial emotion recognition task, eight faces are chosen for each of the six basic emotions from the JAFFE database [43]. Children view the faces on the computer screen and are asked to choose which emotion the face is portraying from a list of the six basic emotions that appear alongside the picture.

##### Cantonese Oral Language Deficiency Early Identification Test for Pre-primary Children (CEIT)

The CEIT is the second locally published standardized language screening test [44]. The contents of the test are mainly selected from the curriculum adopted in Po Leung Kuk kindergartens with additional reference to two local studies on oral language development in preschool children [45, 46]. The test contains 11 sections, which measure a wide range of expressive language knowledge, including vocabulary (nouns, verbs, adjectives, super-ordinates and classifiers), sentence structures (active sentence, passive sentence, comparative sentence, double object construction and relative clauses) and narrative. There are between two and six items in each section and a total of 49 items altogether. Section 1 to 5 (24 items) are selected for use in this study. The test adopts a dichotomized scoring method (i.e. correct and incorrect responses) and children scoring below -1 SD for age are considered to fail the screening [47].

##### Motor automaticity

This test will be administered to assess children’s cognitive-linguistic skills. In the single-task motor automaticity condition, the finger-tapping task and the stepping task will be applied. The finger-tapping task will be adopted from a digital finger-tapping test[48]. Children will be instructed to tap as many times as possible with their index finger of the preferred hand within one minute. The frequency of taps will be recorded. In the stepping task, children are instructed to step on a mini exercise stepper for as many times as possible within one minute. The frequency of steps within one minute will be recorded. In the dual-task coordination, children will be asked to tap as many times as possible with the index finger of their preferred hand while simultaneously stepping on the stepper. The frequency of steps and finger-taps in one minute will be recorded [49, 50].

##### Children’s Color Trails Test (CCTT)

CCTT will be employed as a 2-dimensional paper and pen assessment of visual attention and cognitive flexibility in this study. It is typically used to assess sustained and divided visual attention, sequencing, cognitive flexibility and inhibition [51, 52].

##### M&M (Unexpected contents) false belief test

This test will be administered to assess the first- and second-order Theory of Mind abilities of the children. In the "Unexpected contents" or "Smarties" task, experimenters ask children what they believe to be the contents of a box that looks as though it holds the candy "Smarties". After the child guesses (usually) "Smarties", it will be demonstrated to the childthat the box in fact contains pencils. The experimenter then re-closes the box and asks the child what he/she thinks another person, who has not been shown the true contents of the box, will think is inside. The child passes the task if he/she responds that another person will think that "Smarties" exist in the box, but fails the task if he/she responds pencils [53].

#### Adaptive functioning in real-life situations

##### The Adaptive Behavior Assessment System, Second Edition (ABAS-II)

Will be used to assess adaptive skills where ratings are provided by parents, teachers and caregivers [54]. It is widely used to evaluate independent and social functioning among individuals with ASD. The rating scale measures 10 adaptive behavior skills, including Communication, Community Use, Functional Academics, Health and Safety, Home or School Living, Leisure, Self-Care, Self-Direction, Social, and Motor Skills. In our project, we focus on five of those rating scales, namely communication, community use, leisure, self-direction and social skills.

#### Children’s adherence to the training program

Children’s adherence to this exercise training program will be monitored by their attendance rate of the 16-week training sessions (32 sessions) and their performance record in each session. The performance record for each child will be recorded by the front-line healthcare providers, which includes the items of (1) exercise items the child performed; (2) how many circuits the child finishes in a session; (3) the Borg Rating of Perceived Excretion (RPE) by each child [55]; (4) appearance of physical signs of vigorous exercise (shortness of breath, sweating, face flushing) in each child.

#### Coaching efficacy of the front-line healthcare providers (trainers)

An 8-item adherence score is developed to assess the coaching efficacy of the trainers. The assessment items include whether the trainers (1) can apply simple and direct instruction; (2) have provided visual assisted instruction; (3) have demonstrated the activities; (4) have provided encouragement in the form of small gifts such as stickers; (5) have provided verbal positive reinforcement; (6) completed performance record for children; (7) are able to adjust the tasks accordingly based on the ability of the child; and (8) are able to adjust the tasks accordingly based on the interests of the child (i.e. follow the child’s lead). The adherence score of each trainer will be scored by a professional coach in our research team during his/her visit to the training sessions at the first session and the last session of the program.

#### Evaluation on global functioning improvement of children by their parents

The Clinical Global Impression scale on Improvement (CGI-I) evaluates change from the initiation of treatment on a seven-point scale from 1=very much improved since the initiation of treatment to 7=very much worse since the imitation of treatment [56]. The CGI-I reported by parents or caretaker will be used for evaluating children’s global functioning improvement in three aspects, namely social and communication skills, attention and behaviour and motor functioning.

### Sample size calculation

A priori power analysis was performed in G*Power 3.1 [57] to calculate the sample size required in the present study. The expected difference between the intervention group and the control group was made with reference to the results from a previous social skill training study for ASD individuals [58], in which a difference between the treated and untreated group of 0.90 SD of the mean outcome was yielded. In order to detect clinically significance differences on the outcome measures between the two conditions with 80 % power (α = 0.05; two-sided), 50 children are required per condition. Based on our experiences in past research, we presumed a drop-out rate of 10%, implying a minimal sample size of 50/.9 = 56 children in the control condition and 24/.9 = 56 in the intervention condition (total *n* = 112).

### Statistical methods

Raw data of all key variables will be tested for normality prior to analysis. Analyses will be done using repeated-measures Analysis of Variance (ANOVA) and mixed models as the primary design. Multiple imputations will be used for missing observations at post-intervention and 3-month follow-up. Reporting of the results of the study will be in accordance with the CONSORT 2010 Statement [59].

In the aforementioned analysis plan it is assumed that children will play the game individually and the data will not be clustered. However, in cases of clustered data due to the formation of groups in which several children play the game in the same room, the analyses will be conducted in MPLUS 6.11 [60].

First, the two groups will be compared with respect to age, gender, nonverbal intelligence (IQ), and CAST and AQ-Child severity score in order to assess the comparability between them with independent samples t-tests. Baseline descriptors that differ significantly are taken into account in the following analyses.

To investigate the effectiveness of the TTT program, we explore both within-subject effects (changes within children across time) and between-subject effects (changes between children in the control condition versus the intervention condition). ‘Time’ is the within-subject variable, with three levels: T1 (baseline), T2 (effect), and T3 (follow-up). ‘Condition’ is the between-subject variable, with two levels: intervention condition and control condition. The tasks we employed will all yield continuous data. We corrected for multiple testing using Bonferroni correction. Statistical analyses are performed using SPSS 24.0 statistical software (SPSS) and are two-tailed, with a level of significance of α = 0.05.

### Drop out analyses

For participants in both conditions (the intervention condition and the control condition), we examine the differences between the drop-outs and fully participating individuals concerning several descriptors (age, gender, nonverbal IQ, CAST and AQ-Child severity score) to investigate whether the incidence of drop-outs is selective or random.

## Discussion

The prevalence of Autism Spectrum Disorder is increasing worldwide. However, social and behavioural deficits among children with ASD make interaction with peers difficult, and such physical and social constraints have shown to reduce the physical activity level in children with ASD. Sedentary lifestyle not only affects a child’s general health and family dynamics but may further isolate and deprive the child’s social adaptive function and skills. The design of this protocol is a randomized controlled design, which focuses on young children (4 to 6 years old) with ASD. The protocol also includes a training manual for front-line healthcare providers, which allows them to implement the training to young children with ASD in community settings after they have equipped through the train-the-trainer workshop.

The design of the training program is different from previous studies in that a multidimensional training program which includes a number of training elements is adopted. This allows children to build up their physical conditioning needs for a variety of sports and activities. If this training program is shown to be effective, it can be promoted as one of the interventional strategies among young children with ASD, to equip them life skills which are necessary for enjoying leisure and sport, in turn, enhance physical fitness and psychological well-being. The implementation of the training program by front-line healthcare providers can facilitate the program to be used sustainably in the community.

## Abbreviations

ASD: Autism Spectrum Disorder
TTT: train-the-trainer
NGO: non-governmental organization
